# The Positive Facet of Self-compassion Predicts Self-reported Use of and Attitudes toward Desirable Difficulties in Learning

**DOI:** 10.3389/fpsyg.2017.01353

**Published:** 2017-08-09

**Authors:** Laura K. Wagner, Simon Schindler, Marc-André Reinhard

**Affiliations:** Department of Psychology, University of Kassel Kassel, Germany

**Keywords:** desirable difficulties, self-compassion, self-regulated learning, learning strategies, fear of failing

## Abstract

Previous research found that introducing difficulties and challenges during learning has desirable outcomes. With the present work, we investigated the question how the use of and the attitudes toward such learning strategies (so-called *desirable difficulties*) are related to self-compassion, a concept that describes the tendency to be understanding and kind to oneself when confronted with negative experiences. Evidence suggests self-compassion to be linked to less fear of failing, and further to higher control beliefs and mastery goals in learning. Given that applying desirable difficulties in self-regulated learning implies increased challenges, and further, a higher likelihood to experience a feeling of failing, we expected that the use of desirable difficulties increases with levels of self-compassion. We tested this hypothesis in an online study (*N* = 136) in which self-compassion and the self-reported use of and attitudes toward strategies of desirable difficulties were assessed via respective questionnaires. Results of a correlation analysis yielded first evidence for our idea. Decomposing self-compassion into a positive and a negative facet showed that the positive, but not the negative, facet is positively correlated with attitudes toward and the use of desirable difficulties. Additionally, a regression analysis showed that the positive but not the negative facet predicted attitudes toward and use of desirable difficulties, when entering both facets simultaneously as predictors. Practical implications for learners are discussed.

## Introduction

It is a common assumption that learning should be easy. There is, however, convincing evidence that introducing challenges and difficulties during learning increases desirable outcomes. These so-called desirable difficulties are, for example, emphasized when it comes to optimizing students’ learning strategies in college (Putnam et al., 2016). Given the positive effect of using desirable difficulties in self-regulated learning, the question arises as to which personality traits promote their use. In the present work, we focus on self-compassion (SC), a trait that can be considered as highly relevant for the context of learning because it refers to one’s coping mechanisms when confronted with negative experiences, for example, the fear of failing on a test. Furthermore, research has indicated that SC can be increased by training (e.g., Breines and Chen, 2012), pronouncing the importance of this research for the educational context. It is expected that high levels of SC are positively associated with the use of desirable difficulties.

### Desirable Difficulties in Learning

Desirable difficulties are learning strategies with difficulties and challenges that are beneficial for learners’ outcomes. Increase of learning success is possible because desirable difficulties require more cognitive resources and active processing (McDaniel et al., 2014). Autonomous generation of knowledge, realizing connections, embedding contexts, and the generation of examples and counter-examples are all active processes that are cognitively effortful; however, they can foster learning success (Bjork and Bjork, 2011; Bjork et al., 2013). Desirable difficulties are helpful methods because they lead to encoding and retrieval processes, which enhance learning, comprehension, and the remembrance of learned content.

Most research has focused on five desirable difficulties: (1) self-generation of information, materials, and content, (2) autonomous yielding of a hypothesis and predictions deducted from learning content, (3) testing oneself on learned content, (4) working through exercise materials as mixed form of self-testing and generation, (5) interleaving learning and mixing different themes across time. Research has shown empirical support for the effectiveness of these desirable difficulties. First, investigations have shown that the generation of information leads to enhanced retrieval in comparison to information that is merely presented (McDaniel et al., 1988). Production of predictions results in deeper processing, inferential thinking, and thus better learning than simply reading the answer (Crouch et al., 2004). Active retrieval of the learned themes is further fostered by self-testing (Roediger and Karpicke, 2006). Research has shown likewise that distributed learning and alternating diverse subjects over time improves learning performance compared to when mass practiced (Kornell and Bjork, 2008).

However, desirable difficulties require sufficient previous knowledge and abilities to handle the difficulties successfully (Bjork and Bjork, 2011). Consequently, exhaustive learning is not automatically effective; yet it is required to lead to deeper processing (McDaniel et al., 2014). According to the theory of Lockhart and Craik (1990), the depth of processing influences retrieval. The deeper processing has been, the better learning outcomes will be. Processing information with deep elaboration leads to a connection between new information and pre-existing knowledge.

Given that using desirable difficulties, in fact, leads to desirable learning outcomes, an important question concerns the personality traits that might promote the use of desirable difficulties in self-regulated learning. Learning with desirable difficulties means learning in a way that often requires more cognitive resources. Although research has shown that desirable difficulties have the potential to improve learning outcomes, such learning strategies also imply possible negative effects. For example, self-testing includes the possibility to experience failure, which potentially causes stress and anxiety. Test-anxiety as a state of unpleasant emotional arousal inhibits learners’ performance and therefore must be managed (e.g., Zeidner and Matthews, 2005). Additionally, generating one’s own material and information is a vehement challenge. When a learner is not able to handle this (desirable) difficulty, it seems plausible that a feeling of failure arises. Furthermore, spacing increases the probability that deficits will be uncovered because learned themes that are not in long-term memory often cannot be retrieved later. Accordingly, we assume that using desirable difficulties implies the potential of being confronted with one’s own personal deficits whenever learners are not able to find the right solution immediately, which causes threat to one’s self-concept and results in worse performance (Schmader et al., 2008). Thus, given the possible challenges when using desirable difficulties, their use should be more likely when learners treat themselves kindly rather than judging themselves for one’s mistakes.

### Being Kind to Oneself: The Concept of Self-compassion

Self-compassion means the general tendency to be understanding and kind to oneself when confronted with negative experiences (Neff, 2003b). Specifically, the concept SC incorporates six dimensions (Neff, 2003a): self-kindness, common humanity, and mindfulness reflect the *positive facet*; whereas critical self-judgment, isolation, and over-identification reflect the *negative facet*.

*Self-kindness* refers to being supportive, caring, and understanding to oneself, especially in situations that involve failure and painful experiences. Self-kindness excludes harsh *self-judgment*, which refers to self-criticism and the denial of one’s present experiences (e.g., thoughts, feelings). *Common humanity* refers to the idea that all suffering, failure, and inadequacies are part of the human existence, and that by sharing this fate, all humans therefore are connected. This belief excludes feelings of *isolation* when negative experiences happen to individuals. *Mindfulness* refers to a state in which present (negative) experiences are merely observed from a meta-perspective and are faced in a non-judgmental and non-reactionary manner. Such a state excludes *over-identification* and rumination about thoughts and emotions.

Whereas some studies have focused on interventions to increase SC, most of the research on SC has measured individual differences by using the SC scale (Neff, 2003a) overall, showing a positive relation to life satisfaction, happiness, adaptive coping strategies, empathy, and altruism (Barnard and Curry, 2011; Neff and Dahm, 2015). Investigating the role of SC within the context of learning and performance has revealed that trait SC is positively associated with achievement goals and adaptive coping with academic failure (Neff et al., 2005). Specifically, Dweck et al. (1995) found that people with high trait SC manage failures in a more self-serving way, have less fear of failure, and show less self-conviction after failure. Furthermore, they are more concentrated on following tasks leading to better performance. Additionally, positive correlations were found for SC and higher self-efficacy and higher control beliefs for learning, respectively (Iskender, 2009), and a stronger focus on mastery goals (i.e., to be curious and interested in new learning topics), but a weaker focus on performance goals (comparing oneself with others; Akin, 2008). Finally, experimentally enhanced SC increased self-improvement motivation after receiving information about one’s mistakes (Breines and Chen, 2012).

## The Present Research

Previous research found that introducing difficulties and challenges during learning has desirable outcomes (e.g., McDaniel et al., 2014). With the present work, we investigate the following question: To what extent are the use of and attitudes toward such learning strategies correlated to SC? The concept of SC describes the general tendency to be understanding and kind to oneself when confronted with challenges and negative experiences (Neff, 2003b). Evidence has suggested that SC is linked to less fear of failing, and further to higher control beliefs and mastery goals in learning. Given that applying desirable difficulties in self-regulated learning implies increased challenges and a higher likelihood to experience a feeling of failing, we expect that the attitudes toward and the use of desirable difficulties increase with levels of SC. The latest findings on the factor structure of the SC scale suggest a two-factor solution (López et al., 2015; Pfattheicher et al., 2017). In line with these findings, we test our hypothesis by decomposing SC into the positive and the negative facet. We hypothesize the positive (negative) facet to be positively (negatively) linked to attitudes toward and the use of desirable difficulties. In the following study, SC and the self-reported use of and attitudes toward strategies of desirable difficulties were assessed.

## Materials and Methods

### Participants

Participants in this Internet study included 136 German students (115 females, 21 males; *M*_age_ = 22.59 years, *SD* = 5.12, range: 18–46), recruited via the university’s internal online study platform. Here, all ongoing studies are listed, so that interested students can easily decide where they want to participate. Additionally, the platform enables a quick course credit allocation for psychology students. As is typical for conducting studies online via this platform, a link to the study was provided. Most participants were psychology students (*n* = 87). Other subjects were law (*n* = 4), teaching (*n* = 8), natural science (*n* = 7), sociology (*n* = 7), business (*n* = 4), informatics/mathematics (*n* = 1), and political science (*n* = 1). 17 participants reported “other.” The date was collected within 2 weeks with an online software named questback. Participation lasted about 15 min, was voluntarily and psychology students received college credit for participating.

The study was conducted in full accordance with the Ethical Guidelines of the German Association of Psychologists (DGPs) and the American Psychological Association (APA). Moreover, by the time the data were acquired it was also not customary at Kassel University, nor at most other German universities to seek ethics approval for simple studies on personality and attitudes. The study exclusively makes use of anonymous questionnaires. No identifying information was obtained from participants. Every participant had to read and to agree to the informed consent. They were explicitly informed that the data are treated confidentially and that they may withdraw from the study at any time without explanation. Moreover, it was possible to easily withdraw from the study at any time by closing the internet browser.

### Procedure and Material

After the informed consent was obtained from the participants, they were provided with the SC scale. After some distractor items about implicit theories of intelligence and morality (Dweck et al., 1995), use of and attitudes toward strategies of desirable difficulties were assessed. Finally, participants indicated demographic information. As a measure of respondents’ academic competence, they were asked to report their high school graduation grade (*M* = 2.01, *SD* = 0.67; 13 participants did not indicate a grade).

#### Self-compassion Scale

To assess individual differences in SC, participants responded to 26 items of the German version of the SC scale (Hupfeld and Ruffieux, 2011). Sample items of the positive facet (13 items in total, Cronbach’s α = 0.87) read “I try to be loving toward myself when I’m feeling emotional pain” (self-kindness), “I try to see my failings as part of the human condition” (common humanity), and “When I’m feeling down, I try to approach my feelings with curiosity and openness” (mindfulness). Sample items of the negative facet (13 items in total, Cronbach’s α = 0.86) read “I’m disapproving and judgmental about my own flaws and inadequacies” (self-judgment), “When I fail at something that’s important to me, I tend to feel alone in my failure” (isolation), and “When something upsets me, I get carried away with my feelings” (over-identification). Scale endpoints were labeled as *almost never* (1) and *almost always* (5).

#### Desirable Difficulties Measurement

We assessed the use of and attitudes toward predominant types of desirable difficulties likely adapted in students’ self-regulated learning. These desirable difficulties are represented by two attitude items and one item capturing its self-reported use: (1) self-generation of information, learning materials and content (e.g., “I like to create my own learning materials,” “Compared to other learning methods, I favor working out the subject matter myself”), (2) autonomous production of a hypothesis and predictions deducted from the learning content (e.g., “I enjoy predicting solutions,” “Prior to looking up a solution, I generate the answer myself”), (3) self-testing of learned contents (e.g., “It is fun to test myself,” “I always use opportunities to test my learning success”), (4) working through exercise materials as a form of self-testing and generation (e.g., “It is practical to use available exercise opportunities,” “I utilize exercises for my exam preparation”), and (5) interleaved learning of shuffled topics with differing learning content spread across time rather than block-wise pairing and mass practice (e.g., “I think it makes economic sense to shuffle several topics when it comes to my learning success,” “When I study, I mix topics in a targeted manner and spread them across several days”). These Items were used in a study by Weissgerber et al. (unpublished). Scale endpoints were labeled as *totally disagree* (1) and *totally agree* (7). Items on attitudes toward desirable difficulties (Cronbach’s α = 0.78) and items on self-reported use (Cronbach’s α = 0.63) were compiled into two parts and analyzed separately.

### Data Analysis

We analyzed the data with IBM SPSS Statistics 22. All significance tests are two-sided with a Type I error rate of 5%. We used bivariate correlation analyses based on Pearson’s *r* and multiple linear regression analyses to test our hypothesis.

## Results

To test our hypothesis, we first computed bivariate correlations based on Pearson’s *r* (**Table 1**). In line with previous research (e.g., López et al., 2015), we decomposed the SC scale into a positive and a negative facet. As expected, results indicated a significant correlation between the positive facet and self-reported use of desirable difficulties, *r* = 0.22, *p* = 0.011, as well as attitude toward desirable difficulties, *r* = 0.20, *p* = 0.020. No significant correlations were found with the negative facet, both *p*s > 0.399. Notably, high school graduation grade did not significantly correlate with any of the assessed constructs, all *p*s > 0.551.

**Table 1:** Correlation of attitudes toward and use of desirable difficulties with predictor variables (*N* = 136).

Variable	1	2	3	4	*M*	*SD*
(1) Self-reported use of desirable difficulties	–				4.78	0.91
(2) Attitudes toward desirable difficulties	0.69^∗∗∗^	–			5.03	0.77
(3) Positive facet of self-compassion	0.22^∗^	0.20^∗^	–		2.92	0.64
(4) Negative facet of self-compassion	-0.07	-0.05	-0.68^∗∗∗^	–	3.43	0.70
(5) High school graduation grade	-0.05	-0.04	0.05	-0.02	2.01	0.67


*Thirteen participants did not indicate a high school graduation grade; those correlations are based on an n of 123. Desirable difficulties measured on 7-point scales, self-compassion on 5-point scales. Bivariate correlation coefficients are based on Pearson’s r, ^∗^p < 0.05, ^∗∗∗^p < .001 (two-tailed)*.

Additionally, we investigated the relationship between attitudes and the self-reported use of desirable difficulties and the positive and the negative facet of SC by using linear regression analyses. The two facets of SC were simultaneously entered as predictors. Results showed that the positive facet significantly predicted self-reported use of desirable difficulties, *b* = 0.45, *SE b* = 0.16, β = 0.31, *p* = 0.008, as well as attitudes toward desirable difficulties, *b* = 0.37, *SE b* = 0.14, β = 0.31, *p* = 0.008. There was, however, no significant prediction of the negative facet on self-reported use of desirable difficulties, *b* = 0.18, *SE b* = 0.15, β = 0.14, *p* = 0.223, nor on attitudes toward desirable difficulties, *b* = 0.18, *SE b* = 0.13, β = 0.16, *p* = 0.166. Levels of significance remained unchanged when controlling for high school graduation grade.

## Discussion

The present research investigated the idea that SC as a personality trait promotes the use of specific learning strategies, named desirable difficulties. Desirable difficulties are characterized by difficulties and challenges that are beneficial for a learner’s outcome. SC is conceptualized as the tendency to be understanding and kind to oneself in hard times. Given that evidence suggests SC to be linked to less fear of failing, and further to higher control beliefs and mastery goals in learning, and given that applying desirable difficulties in self-regulated learning implies increased challenges and also a higher likelihood to experience a feeling of failing, we expected that the use of desirable difficulties increases with levels of SC. According to the latest findings on the factor structure of the SC scale (López et al., 2015; Pfattheicher et al., 2017), we tested our hypothesis by decomposing SC into the positive and the negative facet.

In line with our idea, the positive facet of SC predicted self-reported use of and attitudes toward desirable difficulties. This effect remained unaffected when controlling for the negative facet and for high school graduation grade. The cumulated scale of SC further correlated marginally significantly with self-reported use of desirable difficulties, providing additional evidence for our hypothesis. Unexpectedly, however, the negative facet of SC was not linked to the use of, nor attitudes toward, desirable difficulties. According to the data of Pfattheicher et al. (2017), the negative facet of SC does not explain variance on well-being beyond neuroticism. Given that neuroticism is a rather unspecific concept in the context of learning strategies, the role of more specific concepts (e.g., test anxiety) should be addressed in future research.

Although, it seems plausible to assume a causal influence of SC on the use of desirable difficulties, it is important to note that the present study is only correlative in nature. Although SC is treated as a trait (Neff, 2003a), previous work showed that SC can be learned and also increased by experimental manipulations (e.g., Leary et al., 2007; Zabelina and Robinson, 2010; Breines and Chen, 2012). Future studies should thus apply such a paradigm to provide direct evidence for promoting causal effects of SC.

The knowledge about the causal relationship between SC and the use of desirable difficulties is also important regarding the educational context. If SC causally promotes the use of desirable difficulties, it would be beneficial for students to get trained in SC by (e.g., by their teachers) or to train SC by their own. In sum, the influenceability of SC emphasizes the practical relevance of this concept for successfully applying desirable difficulties in learning, and in the educational context in general. In a first step, we provided evidence for a positive correlational relationship.

## Author Contributions

The basic idea was developed by all three authors. LW and SS wrote the manuscript. LW was responsible for study design, data collection and analyses. M-AR gave important comments on the manuscript.

## Conflict of Interest Statement

The authors declare that the research was conducted in the absence of any commercial or financial relationships that could be construed as a potential conflict of interest.
